# The Evolution of an Innovative Online Task to Monitor Children's Oral Narrative Development

**DOI:** 10.3389/fpsyg.2022.903124

**Published:** 2022-07-27

**Authors:** Amy Scott, Gail Gillon, Brigid McNeill, Alex Kopach

**Affiliations:** ^1^Faculty of Education, Child Well-Being Research Institute, University of Canterbury, Christchurch, New Zealand; ^2^Faculty of Education, School of Teacher Education, University of Canterbury, Christchurch, New Zealand; ^3^Global Office Limited, Christchurch, New Zealand

**Keywords:** automatic speech recognition, language sampling, language transcription, children's speech recognition, oral narrative

## Abstract

Oral narrative abilities are an important measure of children's language competency and have predictive value for children's later academic performance. Research and development underway in New Zealand is advancing an innovative online oral narrative task. This task uses audio recordings of children's story retells, speech-to-text software and language analysis to record, transcribe, analyse and present oral narrative and listening comprehension data back to class teachers. The task has been designed for class teachers' use with the support of SLP or literacy specialists in data interpretation. Teachers are upskilled and supported in order to interpret these data and implement teaching practices for students through online professional learning and development modules, within the context of a broader evidence-based approach to early literacy instruction. This article describes the development of this innovative, culturally relevant, online tool for monitoring children's oral narrative ability and listening comprehension in their first year of school. Three phases of development are outlined, showing the progression of the tool from a researcher-administered task during controlled research trials, to wide-scale implementation with thousands of students throughout New Zealand. The current iteration of the tool uses an automatic speech-recognition system with specifically trained transcription models and support from research assistants to check transcription, then code and analyse the oral narrative. This reduces transcription and analysis time to ~7 min, with a word error rate of around 20%. Future development plans to increase the accuracy of automatic transcription and embed basic language analysis into the tool, with the aim of removing the need for support from research assistants.

## Introduction

### Language Sampling and Oral Narratives in Young Children

Language sampling and analysis is a naturalistic method of evaluating and monitoring children's language performance and development. Language sampling collects “real” language through the recording of children's speech, which can be elicited from a variety of different contexts. This sample can be transcribed “in the moment” or at a time after it has been collected. The sample of language can then be analyzed for multiple language features beyond those that may be elicited in a standardized language assessment. Such analysis can include both micro- (e.g., productivity, vocabulary and grammar) and macro-structural use (e.g., narrative structure and organization) (Costanza-Smith, 2010; Westerveld, 2011).

One commonly used method for eliciting language samples from children is the use of a story retell task, allowing for the evaluation of children's oral narrative abilities. In such a task, children are presented with a telling of a story (usually with accompanying pictures), and then are prompted to retell the story in their own words, thus eliciting the retell. Oral narrative abilities are an important measure of children's language competency and have predictive value for children's later academic performance, particularly reading comprehension (Babayigit et al., 2021). As such, children's oral narrative abilities are of interest to teachers and speech language pathologists alike. This article focuses on the transcription and analysis of language samples collected via a story retell task produced by children in their first year of school.

### Barriers to Use by Teachers and Professionals

Oral language sampling and analysis is an important method for gaining insight into children's oral language development in more naturalistic settings, such as retelling a story or sharing a personal experience. Analyzing children's oral language from such contexts is considered “best practice” in an overall language assessment, particularly for children who may be struggling with their oral language or literacy development. It should play a key role in a speech–language pathologist's (SLPs) assessment practices, but barriers exist which reduce the frequency and accuracy of language sampling implementation. The time investment is consistently reported as the biggest barrier to the routine use of language sampling and analysis by SLPs in clinical practice (e.g., Kemp and Klee, 1997; Westerveld and Claessen, 2014; Pavelko et al., 2016; Klatte et al., 2022).

Two main aspects of language sampling are identified as being the most time-consuming for SLPs—transcription and analysis of the sample. There is now a variety of software available to support the coding and analysis of language samples, including Systematic Analysis of Language Transcripts (SALT; Miller et al., 2012), SUGAR Language (Pavelko and Owens, 2017) and Computerized Language Analysis (CLAN; MacWhinney, 2000). Older estimates suggest it takes 5 min to transcribe every 1 min of speech from a typically developing child (Heilmann, 2010), however, this is being improved through approaches such as SUGAR (Pavelko and Owens, 2017). This approach has demonstrated that clinically valid conversational language samples can be collected, transcribed and analyzed in ~20 min by a trained researcher, however, 15 min of that time is dedicated to transcription and coding, and 2 h of training is required prior to using the approach. While technology and research are advancing in this area, further attention is needed to address the time taken to transcribe, code and analyse language samples for sustainable use in clinical or classroom settings.

Routine and systematic monitoring of children's language provides many benefits to teachers of new entrant/kindergarten children. Understanding children's language competency and gaps provides useful information for classroom planning, but also enables early identification of children at risk. For example, teachers may notice a low number of adjectives and adverbs in their class's language samples, so they may focus classroom teaching on building children's knowledge of these types of words for use in their oral and written storytelling. Further, a teacher might use language samples as a monitoring tool to track language growth, and may refer on to a speech–language pathologist should a student not be making the expected progress between assessment points.

Despite this usefulness, it is not common practice for teachers to routinely and systematically monitor children's oral language (Malec et al., 2017; Cameron et al., 2019). There is a paucity of research exploring teacher use of systematic language sampling in kindergarten/new entrant classrooms, and even less research into the development of technologies to support this. Cameron et al. (2019) report on teachers' desire for tools to assess children's oral language, but time and knowledge are also cited as barriers to use.

To overcome the barriers faced by clinicians and teachers regarding language sampling, technological advances need to be harnessed and implemented to improve the useability of the approaches to language evaluation and monitoring. The aim of this study was to utilize technological advancements in automatic speech recognition systems and develop a language sampling tool to address the barriers to its more systematic use. This paper describes the phases of development and technological aspects of this tool.

### Current State of Automatic Speech Recognition for Children's Speech

Advancements in automatic speech recognition (ASR), also known as speech-to-text, have been considerable in the past decade, with some systems now achieving near-human accuracy under optimal conditions. However, accuracy rates for children's speech are consistently lower than that achieved for adult's speech. Even with models trained specifically for children's speech, word error rates (WER) of 30–60% are still being reported (Yeung and Alwan, 2018; Booth et al., 2020; Lileikyte et al., 2020; Fox et al., 2021). Word error rate is a standard metric for measuring the accuracy of speech-to-text systems. It is calculated by adding the number of errors together (substitutions, insertions, and deletions) and dividing it by the total number of words. A WER of 60% means that the automatic transcription is at least 60% incorrect when compared to the actual speech produced.

While work is progressing to improve the accuracy of ASR systems for children's speech, some barriers remain. Accurate recognition of children's speech is difficult for ASR due to the high levels of variability inherent in children's speech production. This variability decreases substantially as age increases, but the speech of children below the age of 6 years old is particularly challenging for ASR systems (Yeung and Alwan, 2018). This leaves a large window of time in which ASR technology cannot be effectively harnessed for use with young children. A further barrier to improving ASR technology is the difficulty in obtaining large enough child speech datasets to effectively train ASR systems, which is required in order to improve their accuracy and performance.

Some recent studies have explored the applicability of ASR for child's speech to improve utility as a clinical tool (Booth et al., 2020; Lileikyte et al., 2020; Fox et al., 2021).

Booth et al. (2020) attempted to improve ASR for children's speech by training models using adult and children's speech datasets. To do this, they used the openly available CMU Kids Dataset which comprises around 9 h of child speech (5180 utterances) from 76 children aged 6–8 years of age. They also collected an additional 454 utterances from 20 children between grades one and five. All utterances were short, read prompts such as “*A blue butterfly flew by*.” They used this combined data set with an additional adult speech dataset to train models and to explore the potential to develop accurate transcription for child speech using smaller quantities of data. They reported a final WER of 29% for the full set of data, with substantial improvements in WER noted in increasingly older age groups.

Fox et al. (2021) gathered their own small dataset and used an existing ASR system (Google Cloud Speech) to compare the accuracy of this tool with two groups of real-time transcribers (SLPs and trained transcribers). Using 42 short narrative samples from children aged 7;5 to 11;10, they examined the transcription accuracy based on WER, and the reliability of four quantitative SALT metrics, on the groups of transcripts. Their analysis showed superior performance of the ASR system compared to either human transcription conditions, in terms of both accuracy of transcription and overall clinical utility. The ASR-generated scripts also had the highest level of reliability on the four SALT metrics (Total Number of Utterances, Number of Different Words, Total Number of Words and MLU-words), despite a WER of around 30%.

While both Booth et al. (2020) and Fox et al. (2021) showed promising results for clinical applications of ASR for children's speech, some challenges remain with its wider use. The WER of both studies was around 30%, which is higher than desirable for this type of technology. Fox noted that, while this degree of error did not negatively impact the quantitative language metrics, it is not ideal when examining more qualitative measures of language use from narrative samples. Fox concluded the ideal application of the technology at present is the use of ASR to produce an initial transcript, with a follow-up check for accuracy by an SLP or other professional using accompanying audio recordings.

The age of the children in the above two studies may also have had an impact. These studies included participants who were 6 years and above and achieved WERs of around 30%. Given that the WER of ASR is sensitive to even 1 year of age difference (Yeung and Alwan, 2018), the accuracy of ASR is likely to be even lower for children at 5 years of age.

Lileikyte et al. (2020) explored the training of an ASR system in preschool children aged 2.5–5 years old using 15 h of transcribed audio from spontaneous speech. Speech samples were collected in an early childcare facility, rather than in a controlled recording condition, which may have negatively impacted ASR accuracy. Even with model training and attempts to enhance the model through augmentation of the dataset, WER remained at around 60%. It is likely that the higher error rate was due to the young age of participants and the recording context in which the language samples were collected.

Despite significant advances in ASR technology for children's speech, current accuracy rates are unsuitable for automatic transcription of language samples for the purpose of oral language assessment. Clear opportunities exist for further development of ASR technology to enable innovative developments of efficient and accurate oral language analysis tools.

## Context and Innovation

The context of this article surrounds the Better Start Literacy Approach research team and teachers involved in a series of related research projects over a 6-year period. Funding for the projects was drawn from multiple sources, and enabled the development of the Better Start Literacy Approach (BSLA) from a pilot project with seven schools and 141 5-year-old children in 2015-16 (Gillon et al., 2019) to a national implementation with over 700 schools and projected 70,000 children in 2021-23. The Better Start Literacy Approach is an early literacy approach for children in their first year of school (Gillon et al., 2019, 2020, In Press). It focuses on building the critical foundational skills needed for early literacy success, including phoneme awareness, letter–sound knowledge, word reading, vocabulary, oral language and listening comprehension. Embedded in the approach is the use of assessments to monitor the development of these foundational skills in response to teaching, and to identify the key next steps for learning. The focus of this article is the evolution of the story retell task over three phases of development.

### Overcoming the Barriers to Wide-Scale Use of Language Sampling

Research and development underway in New Zealand on an innovative oral narrative tool aims to address the barriers to systematic oral narrative sampling as a monitoring tool for teachers and other professionals. The Better Start Literacy Approach has utilized oral narrative sampling as part of its assessment protocol for over 5 years now. However, as the approach has moved from researcher-led controlled studies to a nationwide implementation trial, the feasibility of gathering, transcribing and analyzing tens of thousands of language samples is beyond the scope of the research team and its funding. This shift in research focus requires innovative thinking in order to retain the usefulness of the oral narrative sampling task, while reducing the time and effort required by researchers and teachers to gather these data.

The task itself is presented as a short story, with an audio storytelling and accompanying illustrations, followed by the images being presented again without audio, and prompts to encourage children to retell the story in their own words. The use of a story retell task comes from years of development by Westerveld and colleagues. The earliest version of this task used a short story entitled “Ana Gets Lost.” This story followed a small girl who lost her parents when they were out 1 day, and a friendly policeman helped her find her way home. This story was used to create databases of language samples of typically developing children aged 4;0 to 7;6, which were integrated into the Systematic Analysis of Language Transcripts (SALT) software (see Westerveld et al., 2004, 2012; Westerveld and Gillon, 2010; Westerveld and Vidler, 2016).

**Figure 1:**
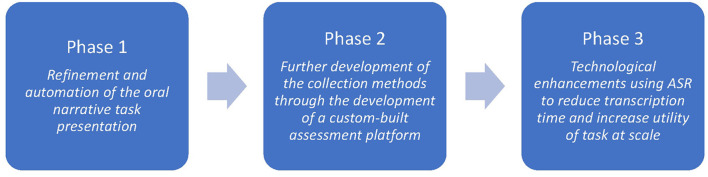
Phases of development of the BSLA oral narrative task.

The development of the oral narrative assessment tool within the Better Start Literacy Approach has occurred in three main phases. Figure 1 shows the three phases and the focus of the development in that phase. These will be discussed in more detail below.

### Phase 1—Refinement of the Oral Narrative Task

Phase 1 of the task occurred as part of a pilot grant for the trial and development of an early literacy approach in Christchurch, New Zealand (Gillon et al., 2019). Initially, the story Ana Gets Lost (Swan, 1992) was trialed as part of the assessment piloting process (see Westerveld and Gillon, 2010). When this story was first trialed with the pilot cohort as part of the co-construction process, feedback from teachers within the community was that children's experience of policeman was not always positive and the story content was not appropriate for all children. In response to this, an alternative story was used. The story, Alice and the Suitcase (Westerveld, 2018), was part of a task created for speech–language pathologists and educators to assess the story retell and comprehension skills of young children. The story was designed to match Ana Gets Lost in terms of length, complexity, semantic diversity and story grammar features. The presentation of the story in Phase 1 consisted of a slide presentation with recorded audio telling the story on each slide. Assessors sat with children, moving through the slides and clicking to play the audio recording of the story. This recorded retell and illustration presentation provided consistency across assessors while being engaging for children.

The protocol for this task consisted of a presentation of the story followed immediately by eight comprehension questions. Children were then asked to listen to the story again, and then retell the story without the support of pictures. The child's retell of the story was captured via a voice recorder which the assessor operated as part of the assessment protocol. Following the audio capture of the child's retell, audio files were downloaded into a shared folder for a transcription team to uplift, manually transcribe, code for language features using SALT software (Miller et al., 2012) and enter data based on language metrics into a spreadsheet. Transcripts were coded following standard SALT coding conventions by a team of speech–language pathology students who completed at least 2 h of training before joining the team of transcribers. The coding covered:

° Comments that were not part of the child's story retell. This included any utterances from the child before they began their retell and anything that came after.
° Unintelligible Segments.
° End of Utterance Punctuation.
° Bound Morphemes.
° Mazes.
° Omissions of words and bound morphemes.
° Error codes at the word and utterance level.

Once coded, transcripts were analyzed in SALT. The analysis and metrics gathered are described in Table 1. SALT analysis provides a wide range of language metrics—those chosen were determined to be of most relevance and use to teachers in an early years classroom setting to guide teaching practice (see Gillon et al., In Press for a comprehensive review of language metrics and data from this task).

**Table 1:** Analysis method and metrics gathered via SALT analysis.

**Analysis method in SALT**	**Metrics gathered**
Standard measures report	Total utterances, % intelligible utterances, MLU in words, Number of Total words, Number of Different words
Grammatical category report	Number of adjectives, nouns, verbs and adverbs

The presentation of the task worked well for the pilot project; however, some adaptions were made to refine it further. For example, while existing literature suggests the removal of pictures generates a more complex language sample in the story retell context (Westerveld and Gillon, 1999/2000), feedback from the pilot was that many children were not providing any language without the support of the pictures, so the decision was made to include the pictures as a retell prompt in the final version of the task. Furthermore, the lead researchers wanted a story more relatable to children from Aotearoa New Zealand. This led to the writing and illustration of a more culturally appropriate story for the New Zealand context. The story, Tama and the Playground (Boston, 2019), was written to emulate the qualitative and quantitative features of Ana Gets Lost and Alice and the Suitcase, with a culturally appropriate and distinctly New Zealand-relevant storyline and characters. Table 2 presents a comparison of the three stories in terms of several linguistic aspects, which were completed through a SALT analysis of the story transcripts.

**Table 2:** Comparison of linguistic features of three story retell scripts.

**Story**	**Ana Gets Lost**	**Alice and the Suitcase**	**Tama and the Playground**
Number of utterances	24	26	25
Mean length of utterance (words)	8	7.46	8.44
Number of total words	192	194	211
Number of different words	108	115	114
Number of nouns	46	50	53
Number of verbs	48	43	43
Number of adverbs	11	12	13
Number of adjectives	6	9	8

**Figure 2:**
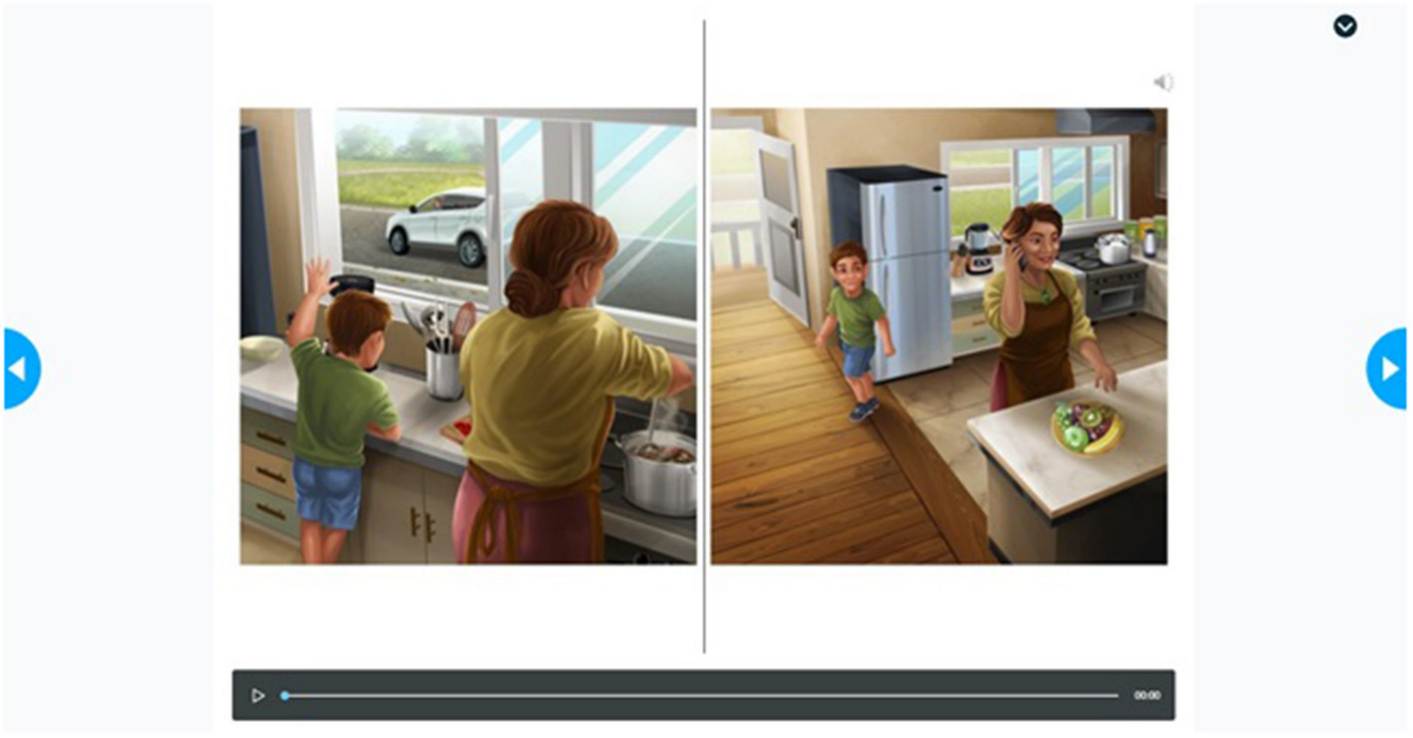
Example of the presentation of the story Tama and the Playground from Phase 1. Reproduced with permission from UC Child Well-being Research Institute.

The final assessment protocol consisted of one presentation of the story, followed by an immediate retell of the story by the child with pictures for support, and finally five comprehension questions. See Figure 2 for an example of the presentation method in Phase 1. Children's responses to the questions were recorded on a paper rubric and later entered into a spreadsheet for data collection. Research assistants completed all tasks in Phase 1.

### Phase 2—Further Development of the Collection Methods

Phase 2 aligned with further advancement of the Better Start Literacy Approach assessment protocol, with a shift to a custom-built website for completing assessment tasks and collecting data on children engaged in research. This development aligned with further funding from A Better Start National Science Challenge and Ministry of Education.

The custom-developed Better Start Literacy Approach assessment platform consists of two web applications—a “Tester” web application, for teachers to undertake testing with students, and an “Admin” web application, for administration of the various features of the website, including user management and task development. Both web applications have been developed using ASP.NET Core (which acts as the API backend) and Angular (which acts as the client-side application). MS SQL Server is used as a database and Azure Blob Storage is used for recorded audio and other media files to be used in assessment tasks. The majority of services and resources for the assessment website are provided by the Azure Cloud Platform from Microsoft.

Within the custom website development, the automatization of the story (Tama and the Playground) was further progressed. The story retell task was now completely embedded within the assessment platform, with the story presentation and audio presented automatically. The audio recordings of children's retells were captured within the assessment website itself using the device microphone, rather than a separate audio recorder. Further, comprehension questions were able to be presented and scored automatically, using a “check box” system. For this, testers read the comprehension questions to children and then selected their answer based on a range of possible responses. The accuracy of the response was scored by the assessment website based on the selection, as either 2 points, for completely correct; 1 point for partially correct; or 0 points for incorrect. These responses were then summed and presented, both as an individual score for each question, and a combined listening comprehension score for the five questions in total.

This streamlining of the presentation, audio recording and comprehension question collection significantly reduced the amount of time and steps required for researchers to collect the language samples. This development saw a shift from research assistants to teachers completing the story retell task with students, taking ~6 min per student to complete.

In Phase 2, the transcription and analysis of language samples was still managed by research assistants employed by the research team. Integration into the custom-built assessment platform meant audio files were able to be bulk-downloaded from the assessment website and transferred into a computerized transcription service (Otter.ai). This transcription service provided an automatic, AI-generated transcript, which research assistants then edited for accuracy within the Otter.ai system. The accuracy of automatic transcription of children's speech was low, and the time and effort required by research assistants to correct the transcription were substantial. Speaker allocation (examiner and child) was also poor, with transcribers needing to manually identify and allocate utterances to each speaker as they edited. The main benefit of the Otter.ai system was the integrated text-editing tool, which was well-developed and allowed for faster transcription of audio files than previously used processes.

Following the completion of the transcription, files were then transferred to the SALT software system (Miller et al., 2012) for coding and language analysis. Coding and analysis followed the same protocol as described in Phase 1 (see section Phase 1—Refinement of the Oral Narrative Task and Table 1). Transcripts were bulk analyzed and a data spreadsheet uploaded to the assessment website, where language metrics were entered into each student's profile, along with the audio file and transcript of the retell. Teachers were able to access this data for their students via the assessment website.

Teachers were supported to interpret this data and implement effective teaching practices for language development through online professional learning and development modules, within the context of a broader evidence-based approach to early literacy instruction (see Gillon et al., In Press for a comprehensive description of the PLD). The modules included the building of teachers' own foundational linguistic knowledge in phonology, morphology, semantics and syntax, as well as their understanding of the language metrics available from the task and how to use this information to identify the next steps for teaching. The modules were accessed through an online, self-directed learning format, and were available to teachers for the duration of the course (12 months). An online teaching session was also delivered via Zoom, to further unpack the content on language metrics and using language data to identify the next steps for teaching.

While Phase 2 developments saw a significant streamlining of the use of this task, the time required for transcription and analysis was still significant, taking ~15–20 min for each language sample. Further work was required to make this task feasible for use by classroom teachers.

### Phase 3—Technological Enhancements to the Automatic Transcription of Language Samples

Phase 3 development was prompted by a research grant from the Ministry of Education to support the nationwide implementation of the Better Start Literacy Approach throughout New Zealand, which is currently underway at the time of preparation of this article. The quantity of children engaging in this implementation will have reached up to 70,000+ by the end of the research funding period. This requires further automation of the transcription of story retell for the task to be sustainable.

Advancement of the oral narrative tool in Phase 3 was focused on the automatization of the transcription and analysis of the language samples, to reduce human interaction to a minimum. To date, over 19,000 samples of the story retell of Tama and the Playground have been collected, allowing for a custom model of transcription to be developed.

#### Development and Training of the Automatic Transcription Service

The automation of the transcription process required engagement with a web development team with specialist skills in automatic speech recognition. The research and web development team worked together to create a system that utilized the latest speech-to-text technology with an interface accessible and functional for teachers.

The technology selected for this work was the Speech-to-Text service from the Speech Service in Azure. This service provides pre-trained models (baseline models) that can be used to train custom models to suit the end user. To train the custom models, for example for the story Tama and the Playground, the development team was able to take advantage of the existing data set collected throughout Phase 2 (which included audio samples and human-corrected transcriptions) to prepare training and testing datasets.

Dataset preparation included breaking language samples into audio “chunks” of a duration considered optimal for training the of models (10 s per audio “chunk”). This includes segmenting the audio into complete phrases (e.g., not part way through a word) that total as close to 10 s as possible. The current models are each trained on ~7,000 audio chunks of around 10 s each. The maximum duration of the audio in the training dataset cannot be longer than 20 h, thus the current approach is to take the latest audio recordings and transcriptions until that limit is reached. The minimum audio duration recommended for training is 30–90 min.

While preparing datasets for training, recorded audio and transcriptions are filtered by various parameters shown by data analysis to differentiate between performance on the oral narrative task. These parameters include student ethnicity (five predetermined categories, or “all ethnicities”); student age group (younger or older than 68 months); number of different words used (greater or <35 different words used); and intelligibility percentage (greater or <90% intelligible).

**Figure 3:**
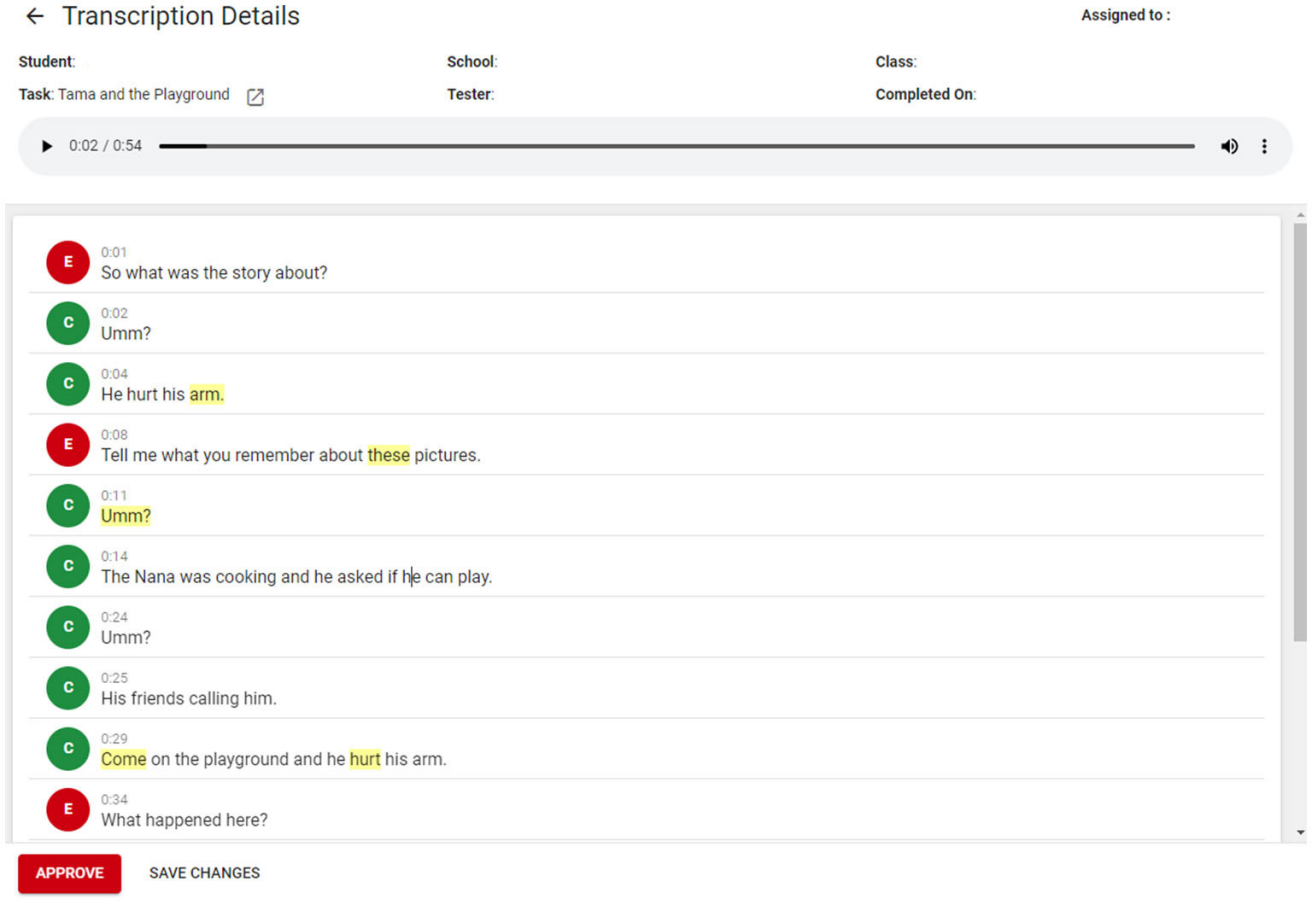
Screenshot from the automatic transcription and editing tool. Reproduced with permission from UC Child Well-being Research Institute.

This initial development phase allowed for the integration of automatic transcription and transcript editing into the assessment website. This meant the child's language sample was now able to be automatically transcribed and then edited for accuracy, within the assessment website itself, rather than pulling the audio file out to be transcribed in an alternative platform (namely, Otter.ai). The transcription interface was developed based on feedback from the team of transcribers regarding useful features of a transcription editing system. These features included keyboard shortcuts to pause and play audio; the use of the arrow key to move with the text as the audio is presented; and the automatic playback of audio from the point where the text is clicked. See Figure 3 for an example of the embedded transcription and editing platform.

#### Impact of Development on Collection and Transcription of Language Samples

In Phase 3, the transcription of language samples was embedded into the assessment website. Children complete the story retell via the assessment website, and within 10 min, an automatically produced transcript was available. Research assistants were still utilized to check the accuracy of the automatic transcription and edit transcripts as required, although the time and effort required for this was greatly reduced. The embedded transcription system uses a color code to identify the confidence level of the automatic transcription for each word. Words highlighted in yellow indicate that the confidence of the automatic transcription based on the custom model is below 80%, and words in red mean it is below 60% (see Figure 3 for example transcript with color coding). Hovering over a word displays the exact confidence level. Transcribers scan the automatically generated transcript, listen to the audio and edit as required. Speaker allocation was also improved in Phase 3. As part of the transcription process, speakers are allocated to lines of automatically generated transcription (Speaker 1 or Speaker 2). Once the automatic transcription is complete, a bank of opening phrases typically used by the examiner allows for identification of which speaker is the child and which is the educator, and lines of transcription are automatically allocated accordingly.

Once transcription of the language sample is complete and approved, a SALT file is automatically generated, pulling relevant demographic data from the assessment system and creating the required file type. Coding and analysis of language samples was completed as per Phase 1 (see section Phase 1—Refinement of the Oral Narrative Task Above and Table 1).

The development in Phase 3 demonstrated a significant reduction in the time required for a research assistant to interact with each transcript, with the total time required for transcription, coding and analysis of a story retell being reduced to around 7 min per sample.

#### Continued Improvement of Transcription Accuracy Through Custom Models

Part of the continued development of the automatic transcription service includes the continued improvement of its accuracy as more language samples are gathered and the automatically generated transcriptions are edited for accuracy by the research team. For this, a similar process to that followed when the initial model was developed (as described in Development and Training of the Automatic Transcription Service) is undertaken.

A set of custom models have been developed based on the stable parameters that the assessment system can gather before the transcription of the language sample is completed (namely ethnicity and student age band). The combination of these parameters creates 12 different models. These models are periodically triggered to a training cycle based on recently collected transcriptions. Once the custom models are trained, an evaluation is completed using the testing dataset and compared with the previously trained model with the same parameters. If the word error rate (WER) of the new model is lower than that in the previous model, a new custom model is published. This improves transcription accuracy for any future transcripts that fit the parameters of that particular model.

Many factors influence WER, including age and accent of the speaker, background noise and audio quality. Table 3 shows the WER for the baseline model (that is, before the automatic transcription has been trained using the children's speech samples) compared to the current custom model. Microsoft reports a WER of 20% as acceptable and 5–10% as good quality (docs.Microsoft.com, 2022). As is shown by the difference between the baseline model and the current model WER in Table 3, substantial improvements in transcription accuracy have been made in this early phase of development. The current model's WER currently ranges from 15.90 to 20.30%. Further improvements in WER will occur as the system trains and evaluates new custom models based on increased numbers of audio samples and corrected transcripts being collected.

**Table 3:** Word error rate for custom models compared to baseline model.

**Custom model parameters**	**Current model word error rate**	**Baseline model word error rate**	**Difference**
All ethnicities, age <68 months	20.03%	53.80%	33.77%
All ethnicities, age > 68 months	20.30%	54.60%	34.30%
NZ European, age <68 months	18.50%	55.10%	36.60%
NZ European, age > 68 months	20.90%	55.60%	34.70%
Māori, age <68 months	20.10%	55.50%	35.40%
Māori, age > 68 months	20.00%	55.60%	35.60%
Pasifika, age <68 months	19.40%	50.01%	30.61%
Pasifika, age > 68 months	19.00%	53.70%	34.70%
Asian, age <68 months	19.00%	50.89%	31.89%
Asian, age > 68 months	19.70%	48.10%	28.40%
Other ethnicity, age <68 months	15.59%	52.10%	36.51%
Other ethnicity, age > 68 months	15.90%	48.70%	32.80%

## Discussion

This case study presented the evolution of an innovative online monitoring tool for children's oral narrative development. Through its three phases of development, substantial progress was demonstrated in the useability of the task as a large-scale research tool, but also in its practicality and usefulness as a classroom assessment tool. Streamlining, and thus reducing the time taken to collect, transcribe and analyse language samples increases the feasibility of routine use of language sampling for SLPS and teachers.

The harnessing of current technological advances in ASR can help address the time required to transcribe language samples of children. However, current research shows limitations in the capacity of custom automatic transcription models to reach a level of accuracy adequate for use. WERs in recent studies are reported as between 30 and 60%; a rate not considered functionally useable (Booth et al., 2020; Lileikyte et al., 2020; Fox et al., 2021). In stark contrast, the current study described the development of custom transcription software and models based on a large dataset of children's story retells, which are currently achieving WERs of 15–20%. Even more noteworthy is that these WERs are achieved in samples of children aged 5–6 years old, a notoriously challenging age group for ASR technology (Yeung and Alwan, 2018).

While more research into systematic teacher use of online oral language sampling is warranted, the advancements made in the development of this tool offer promise for its use by teachers, SLPs and other professionals. The barrier of time, which is frequently cited in the literature, has been addressed in a number of ways. First is the online presentation and collection of oral narrative samples using the custom-built Better Start Literacy Approach assessment platform. This allows the completion of a story retell task and accompanying comprehension questions to be completed in around 6 min per child. Secondly, the development of custom ASR models allows for automatic transcription of the language sample. With minimal input required from research assistants to check accuracy of the automatically generated transcript, the transcription and analysis of the language samples is completed in around 7 min. This takes the total time to collect, transcribe, code, and analyse an oral narrative language sample of 5–6-year-old children to around 13 min in total. Future development of the task aims to achieve automatic transcription accuracy at a high enough level that human edits are minimal, along with integration of basic language analysis measures within the assessment tool. With the barriers to time and use addressed, a future with systematic language sampling forming part of everyday classroom assessment practices looks very feasible.

### Future Directions

Phase 3 represents an exciting step toward the wide-scale, independent use of an oral narrative task by teachers in Aotearoa, New Zealand. Future directions for this task are currently in development. The main focus of Phase 4 will be the improved accuracy of automatic transcription through the continued updating of the custom models, with an aim of having teachers checking and editing children's story retell transcripts themselves following the completion of the oral narrative task. Further development also aims to include the basic analysis of language features such as number of words, number of different words and number of items in certain grammatical categories, without the need to use SALT for coding and analysis.

The intention of Phase 4 is an eventual shift to teachers completing the story retell task, checking and making minor edits to automatically generated transcripts, and determining if further analysis of the language sample is required. This would then be flagged to the research team, who would undertake detailed coding and analysis of language samples for children who required further exploration and monitoring. The goal is for the completion of the assessment task with the child, and the follow-up work required by the teacher, to fall within a 10-min time period.

## Data Availability Statement

The original contributions presented in the study are included in the article/supplementary material, further inquiries can be directed to the corresponding author/s.

## Author Contributions

AS developed the concept and wrote the first draft of the manuscript. GG and BM provided edits to the draft. AK wrote the technical sections of the manuscript. All authors contributed to the manuscript revision, read, and approved the submitted version.

## Funding

Multiple grants contributed to the development of this assessment tool. They include A Better Start National Science Challenge from Ministry of Business Innovation and Employment (New Zealand) (Grant No. 15-02688) and several grants from the Ministry of Education (New Zealand). We would also like to recognize the support of the University of Canterbury's Child Well-being Research Institute for its funding support.

## Conflict of Interest

AK is employed by Global Office Limited, New Zealand. The remaining authors declare that the research was conducted in the absence of any commercial or financial relationships that could be construed as potential conflicts of interest.

## Publisher's Note

All claims expressed in this article are solely those of the authors and do not necessarily represent those of their affiliated organizations, or those of the publisher, the editors and the reviewers. Any product that may be evaluated in this article, or claim that may be made by its manufacturer, is not guaranteed or endorsed by the publisher.
